# Differentiation of Pancreatic Cyst Types by Analysis of Rheological Behavior of Pancreatic Cyst Fluid

**DOI:** 10.1038/srep45589

**Published:** 2017-03-30

**Authors:** Iyad Khamaysi, Aiman Abu Ammar, Gleb Vasilyev, Arkadii Arinstein, Yehuda Chowers, Eyal Zussman

**Affiliations:** 1 Department of Gastroenterology, Bruce Rappaport Sch Med, Technion IIT, Haifa 32000, Israel; 2 Gastroenterology department, Rambam Health Care Campus, Haifa 31096, Israel; 3 NanoEngineering Group, Faculty of Mechanical Engineering, Technion – IIT, Haifa 32000, Israel

## Abstract

Differentiation between mucinous and non-mucinous pancreatic cysts is exceedingly important and challenging, particularly as the former bears malignant transformation potential. Pancreatic cyst fluid (PCF)-based diagnostics, including analyses of biochemical markers, as well as cytology, has shown inadequate accuracy. Herein, a preliminary single-center study of 22 PCF samples, collected by endoscopic ultrasound-guided fine needle aspiration (EUS-FNA), assessed the rheological behavior of PCF and its correlation with lesion type. The dependence of PCF shear viscosity on shear rate was found to follow a power law and could be fitted using Ostwald–de Waele model. Three types of flow curves were identified, where two types correlated with non-mucinous cysts, differing by their power law exponent, and the third type corresponding to mucinous cysts. Viscosity measured at a high shear rate was shown to serve as an accurate and independent marker distinguishing between mucinous and non-mucinous cysts, with an optimal cutoff value of *η*_*c*_ = 1.3 *cP* The accuracy of this novel technique proved superior to string-sign, cytology, carcinoembryonic antigen, and amylase assessments. Moreover, the combined predictive value of *η*_*c*_ and patient age provided for sensitivity and specificity of 100% and 95.5%, respectively. This simple and rapid diagnostic tool can be immediately implemented after EUS-FNA sampling.

Pancreatic cancer is the fourth leading cause of cancer deaths among men and women, accounting for 6% of all cancer-related deaths, with a collective median survival time of 4–6 months. Pancreatic cancer is difficult to diagnose in its early stages; at the time of diagnosis, 52% of patients have distant metastases and 26% present regional spread^1^. Cystic lesions of the pancreas, a harbinger of pancreatic cancer, remain one of the most challenging lesions to diagnose and treat. Such cysts are frequently incidentally detected in patients undergoing abdominal imaging [i.e., computed tomography (CT) or magnetic resonance imaging (MRI)] for other indications. The most commonly occurring pancreatic cysts lack malignant potential, including retention (simple) cysts, pseudocysts and serous cystadenomas (Fig. 1). Less common are mucinous cystic neoplasms (MCNs) and intraductal papillary mucinous neoplasms (IPMNs) that have malignant potential and require surgical intervention. Some lesions, such as serous cystadenomas or pseudocysts, can be monitored, while others, such as cystic pancreatic adenocarcinomas and neuroendocrine tumors, must be promptly resected. Still more complex is the treatment of MCN, which, depending on a variety of characteristics, may or may not need to be resected^2^,^3^,^4^. Owing to the possibility of malignancy, its immediate impact on patient survival and oftentimes urgency of active intervention, there is a need to differentiate between benign and malignant pancreatic lesions. At the same time, unnecessary pancreatic operations bring with them a high mortality rate, which can be avoided by accurate diagnosis.

Imaging plays a vital role in both detection and characterization of pancreatic cyst lesions. An example of representative CT, MRI, and endoscopic ultrasound (EUS) images of pancreatic body cysts, are presented in Fig. 2. CT, MRI, and EUS can provide a clear indication of cyst location and morphology, but still suffer from inadequate diagnostic accuracy^5^. The endoscopic ultrasound-guided fine needle aspiration method (EUS-FNA) allows for aspiration of pancreatic cyst fluid (PCF) from suspected lesions^5^,^6^, which, upon analysis, can enhance diagnostic sensitivity and can be a helpful tool in distinguishing between benign and malignant pancreatic lesions^7^.

PCF analysis for cytology and markers has been widely used for the differential diagnosis of pancreatic cysts^8^,^9^,^10^,^11^,^12^. Among them, the carcinoembryonic antigen (CEA) marker demonstrates the highest diagnostic accuracy in discriminating between mucinous cyst (MC) and non-mucinous cyst (NMC)^13^,^14^,^15^, and in a large multicenter study^16^, demonstrated 77% specificity and 61% sensitivity at a cutoff value of 192 ng/mL cyst fluid CEA. However, CEA levels cannot accurately differentiate between pancreatic cyst types, and therefore is not used as a stand-alone diagnostic measure^17^. Cyst fluid amylase is a useful marker, as it is elevated in pseudocysts and cysts, such as IPMNs, that communicate with the pancreatic duct^18^. The rheological characteristics of the PCF can serve as an indirect measure of the concentration and structural properties of cyst fluid mucin, glycoproteins and DNA content^19^. A common method for the characterization of PCF is the ‘string-sign’ method, which measures PCF properties under rapid extension^2^. The method involves placing a sample of the aspirated fluid between the thumb and index finger and quickly separating them to measure the distance between the fingers before the sample breaks. It was reported that a higher break length of the cyst fluid correlates with a lower likelihood of a benign cyst, while a 1 mm increase in break length corresponds to a 116% increase in the likelihood of a mucinous cyst^2^. This feature may be the result of loss of elasticity in the fluid, or disentanglement of the protein network as a result of the capillary pressure in the thread, which tends towards very high values^20^. However, the method lacks a theoretical framework for predicting the viscoelastic nature of the cyst fluid, which can typically be characterized by the viscous and elastic response of a fluid under deformation^21^,^22^. The viscous component relates to energy dissipated during flow, while the elastic component relates to energy stored during flow, both of which can be altered by variations in the PCF structure or composition. As an example, it was demonstrated that introduction of a small amount of protein (e.g., 0.01% w/w) into a Newtonian medium can lead to a drastic change its viscous response, transforming to yield behavior, which is typical for structured liquids^23^. Also, increasing the concentration of mucins in gallbladder bile fluid resulted in increased viscosity^24^. An additional form of analysis performed on cyst fluids involves measurement of the relative viscosity of the aspirate under shear flow through a capillary. The relative viscosity of fluid from benign cysts has been shown to be significantly lower than of fluids derived from mucinous and malignant cysts. The relative viscosity of cyst fluids demonstrated high values (>1.63) in 89% of mucinous tumors and low values in all tested fluids from pseudocysts and serous cystadenomas. Relative viscosity values below the 1.63 cut-off accurately predicted non-mucinous cysts and strongly suggested that the cyst was benign^15^. In a more recent study, it was shown that relative viscosity was significantly higher for MCNs compared to pseudocysts and serous cystic tumors (1.8–1.9 and 1.2–1.3, respectively)^25^. Thus, the viscosity of the cyst fluids appears to be strongly affected by the nature and relative ratios of the dissolved constituents, which differ between cyst types. The aim of the present study was to assess the utility of the rheological properties of pancreatic cyst fluid, as compared with cytological and biochemical analyses, in differentiating between pancreatic cyst types. The proposed approach is based on measurement of the shear viscosity of PCF and construction of a flow curve of the cyst fluid. Furthermore, it was shown that the combination of several markers, such as viscosity and age of patient, increased diagnostic sensitivity, specificity and accuracy, and bear prognostic value and factors to integrate in clinical decision-making guidelines.

## Results

A total of 22 patients (mean age: 57.1 years [SD = 16.2], Table S1) underwent EUS-FNA; cyst fluids were sent for cytological, biochemical and rheological assessments. Cysts were classified as MC (mucinous cystadenoma, mucinous adenocarcinoma, IPMN) or NMC (serous cystadenoma, pseudocyst), based on surgical and/or clinical findings (presentation, follow-up, imaging and fluid analyses). The baseline patient characteristics, the diameter of the cyst, the location of the cyst in the pancreas, the level of tumor markers (CEA, amylase), results of the cytological examination, string sign and rheological measurements are presented in Table 1. Overall, 10 lesions (45.45%) were classified as MC, while 12 (54.54%) were classified as NMC, 5 of which (22.72%) were considered pseudocysts.

For the rheological assessment, the extracted fluids were evaluated by both string-sign test and viscosity measurements. Flow curves were drawn up, with the viscosity, *η*, plotted against the shear rate, *γ*. In order to describe PCF behavior across the range of shear rates, a power law model was employed:


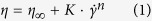


where, *K* and *n* are the fitting parameters and *η*_*∞*_ is treated as infinite viscosity.

**Figure 3:**
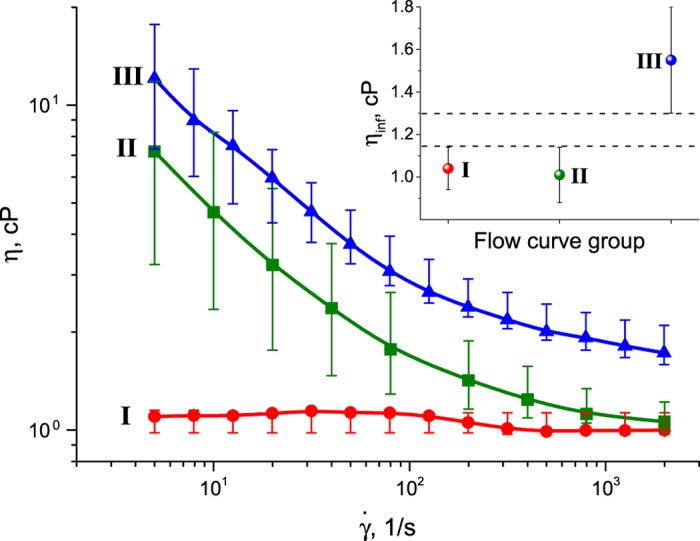
Representative types of I, II and III flow curves. The graph inset shows the values of infinite viscosity, *η*_*∞*_, depicting the difference between the minimal value of type III and the maximum value of types I and II.

This power law, also known as the Ostwald–de Waele power law, is typically used to describe the behavior of non-Newtonian fluids. For example, if *n* < 1, the power law predicts that the apparent viscosity will decrease indefinitely with increasing shear rate. Fitting of the flow curves of the PCFs using Eq. 1, and clustering the fitting parameters (*K, n* and *η*_*∞*_) (Table 2), resulted in three types of flow curves (I, II, III) (Fig. 3). More detailed information, including flow curves, as well as the values of *K, n* and *η*_*∞*_ for each of the tested PCF samples, is provided in the Supplementary data (Figure S1 and Table S2, respectively). At infinite shear rate, 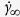, type II and I flow curves demonstrated nearly similar low viscosity, *η*_*∞*_ = 1.01 *cP* and *η*_*∞*_ = 1.04 *cP* respectively, while a significantly higher infinite viscosity, *η*_*∞*_ = 1.55 *cP*, was found for type III. In addition, at low shear rates, the viscosity of type III samples was one order of magnitude higher than the type I samples, and double that of the type II samples, indicating an entirely different fluid micro-structure. Based on the diagnostic results determined using clinical findings (Table 1), we can induce that NMCs display types I + II rheological behavior, while MCs display type III flow curves. Statistical analysis of the flow curve classifications (Table 3) demonstrated a significant difference in the rheological behavior of MCs (flow curve III) versus NMCs (flow curves I + II) (p < 0.0001). It is evident in the inset of Fig. 3, that confidence intervals for flow curve of type III and that of the types I + II do not overlap, and the difference between the minimal value of infinite viscosity of group III and the maximum value of *η*_*∞*_ of types I + II, marked by dash lines, covers the range of *η*_*∞*_ = 1.15−1.30 *cP*. These findings indicate that *η*_*∞*_ is a potentially suitable marker for distinguishing between MC and NMC. However, infinite viscosity is an extrapolated value, thus, it was decided to measure the viscosity, *η*_*c*_ at high shear rates (i.e., 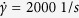); they proved similar to those determined at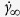.

For the purpose of comparing between predictive factors in their diagnostic capacity to differentiate between mucinous and non-mucinous PCFs, the statistical significance of mean values measured for flow curve types I + II versus III are presented in Table 3. It can be seen that CEA levels were significantly lower among NMCs as compared to MCs (p < 0.014). No significant difference in amylase levels was observed between MC versus NMC, although they increased significantly in pseudocysts, compared to other NMCs (Table S1). It is important to mention that patients with MCs were older than those with NMCs (69.4 and 46.8 years old, respectively). Moreover, there was no substantial difference in cyst diameters between MC and NMC lesions. However, mean viscosity *η*_*c*_ values were significantly different between type I and II lesions versus type III lesions (p < 0.0001).

**Figure 4:**
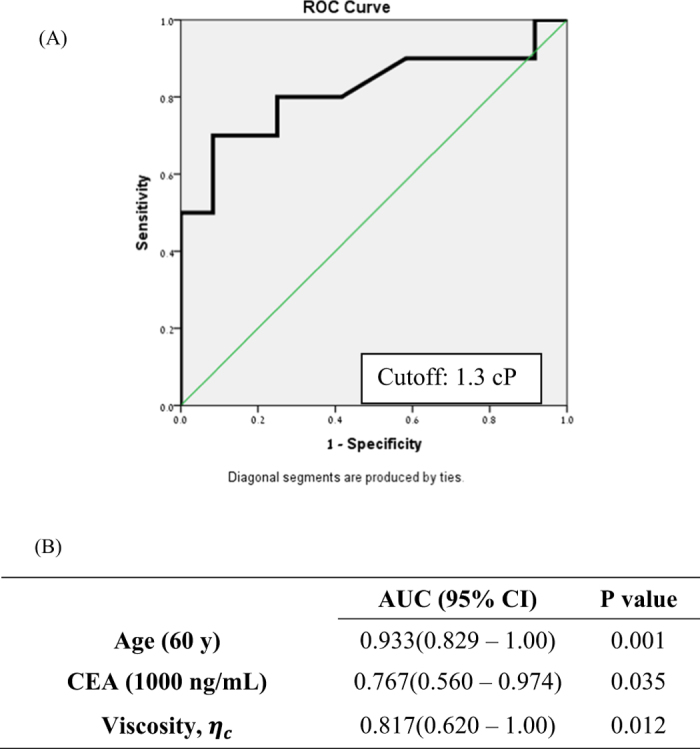
(**A**) Receiver operator characteristic (ROC) curve evaluating the performance of cyst fluid viscosity, *η*_*c*_, measured at strain rate 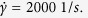
(B) ROC analysis of age, CEA and *η*_*c*_ in differentiation between MCN vs. NMC.

Receiver operator curve analysis of the cyst fluid viscosity, *η*_*c*_, established that the optimal cutoff value for differentiating between MC (flow curve III) versus NMC (flow curve I and II) cysts was 1.3 *cP* (Fig. 4A), with area under the curve (AUC) of 0.817 (Fig. 4B). The sensitivity, specificity and accuracy of cystic fluid CEA, amylase, cytology, string sign, age, viscosity *η*_*c*_ and various combinations of these criteria used to predict whether the cysts were MC or NMC, are presented in Table 4. Using the optimal cutoff value, the sensitivity, specificity, and accuracy of cyst fluid viscosity *η*_*c*_ - based diagnosis of MC versus NMC were 70%, 91.7%, and 81.8%, respectively. In comparison, string-sign analysis showed a sensitivity, specificity and accuracy of 50%, 66.7%, and 59.1%, respectively. The overall accuracy of the viscosity-based technique (81.8%) was greater than that of CEA (72.7%), amylase (50%), cytology (72.7%) and string-sign (59.1%). When considering cyst fluid viscosity, *η*_*c*_ jointly with patient age, the sensitivity and the accuracy increased to 100% and 95.5%, respectively, but the specificity remained 91.7%.

## Discussion

When focusing on the change in viscosity, *η* as a function of shear rate, 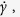 three types of flow curves were easily distinguished (Fig. 3). For type I fluids, the viscosity was constant at all shear rates, close to Newtonian behavior, *n*∼0, where the absolute values of the viscosity were close to viscosity of water at room temperature. This behavior is typical of low molecular weight fluids as well as for diluted polymer solutions or dispersions. Types II and III PCF samples demonstrated yield behavior *n* < 0, i.e., strong shear thinning at low shear rates. This kind of behavior is typically associated with the presence of a structure in the fluid (e.g., formed by proteins), which is destroyed upon application of the shear field, resulting in a sharp drop in viscosity. The viscosity tends to level off, approaching a constant value (infinite viscosity, *η*_*∞*_) as the shear rate increases. The lowest constant viscosity reflects the properties of the fluids when its structure has been completely destroyed (disentangled macromolecules). Such behavior is expected of a multicomponent fluid, such as PCF, whose bulk is comprised of mucins, characterized by high molecular weight, heavily glycosylated proteins^4^.

It has been established that MCs show elevated concentration of mucins in comparison with NMCs^25^, corresponding to flow type III. Indeed, the present analysis classified the rheological behavior of PCFs from malignant or potentially malignant lesions as type III, which was later corroborated by surgical pathology. In addition, no correlation was observed between the diagnostic capacity of the viscosity, *η*_*c*_, measurements to that of the CEA and amylase. For instance, viscosity evaluations, as well as pathology, categorized PCF samples nos 5, 15 and 21 as MC, while CEA and amylase levels of samples nos 15 and 21 differed from sample no. 5 by four orders of magnitude (Table S3). Moreover, PCF sample no. 1 displayed a high CEA level of 531 ng/ml and was therefore considered a MC, yet, surgical pathology classified this tumor as serous (Table S3) and the rheological behavior of this sample matched that of flow curve type I. These results underscore the wide variability and suboptimal accuracy of CEA-based diagnosis, as well as the need for alternative and more precise diagnostic tools^17^.

The rheological properties of PCF are strongly influenced by mucin content, which is commonly manually evaluated, by means of the subjective and variable string-sign method. In their examination of the value of string-sign determination in differentiating between pancreatic cyst types, Leung *et al*. found that increased cyst fluid viscosity was associated with malignant or potentially malignant cysts^2^. In a recent study, Bick *et al*.^26^ evaluated the utility of the string-sign approach in the diagnosis of mucinous pancreatic cysts; high specificity, as well as improved diagnostic accuracy of the method was mentioned. The authors considered the test results positive when the string was at least 1 cm long and remained stable for at least 1 second before disruption, by subjective judgment. Our analysis of the flow curves of the cyst fluids, exhibited better performance than the string-sign method, as manifested by sensitivity, specify, PPV, NPV and accuracy values (Table 4). Moreover, when used as a sole parameter, measurement of viscosity, *η*_*c*_, at a high shear rate (20001/*s*, was superior to other methods, with an overall accuracy of approximately 81.8%. Furthermore, when combined with age, the method provided for impressive sensitivity and accuracy of 100% and 95.5%, respectively. Utilization of this approach provides for robust results, with less variation compared to the string-sign method, due to the use of a rheometer instead of subjective assessments.

## Conclusions

Rheological characterization of pancreatic cyst fluid proved a promising and simple means of identifying potential pancreatic malignancies. Three distinct flow curves of the rheological behavior of PCFs were identified, with types I and II hypothesized to correlate with non-mucinous cysts, and type III with mucinous cysts. The cutoff value of viscosity, *η*_*c*_, measured at strain rate 20001/*s*, can serve as an independent marker to distinguish between mucinous and non-mucinous cysts. It was found that *η*_*c*_ > 1.3 *cP* characterizes MCs, whereas *η*_*c*_ > 1.3 *cP* is typical for NMCs. This simple and rapid diagnostic tool can be immediately implemented after EUS-FNA sampling, and provides for a low variability rate compared to the commonly used, subjective string sign technique. Although the findings are promising, they must be further confirmed in a large-scale study.

## Methods

### Ethics statement

All experimental protocols were approved by the Rambam Medical Center Ethics Committee (0064-14-RMB, 2^nd^ February 2014) and were carried out in accordance with the approved guidelines. All patients signed an informed consent form prior to the EUS-FNA or blood sampling.

### Study design and patients

The cohort consisted of 22 (11 males and 11 females) patients with a suspicious pancreatic cystic lesion, detected by cross-sectional imaging (CT and/or MRI scan) and consequentially referred for an EUS-FNA. Pancreatic cyst fluid samples were collected for cytological and biochemical analysis and patients were followed-up. In addition to routine cyst fluid analysis (biochemical, cytological and string-sign viscosity assessment), rheological measurement were performed (see below).

Pancreatic cysts were classified as mucinous (mucinous cystadenoma, mucinous adenocarcinoma, IPMN) or non-mucinous (serous cystadenoma, pseudocyst), based on surgical and/or clinical findings (presentation, follow-up, imaging and fluid analysis).

### Cyst fluid collection and evaluation

A curvilinear array echoendoscope (Pentax Inc. EG-3630U, Montvale, NJ) was used to insert a 22-gauge needle (Cook Medical Inc. Bloomington, IN, Boston expect or Olympus Inc.), under ultrasound guidance, to aspirate the cyst fluid. Cyst fluid characteristics were recorded. The levels of biochemical markers of cyst fluid CEA and amylase were measured using commercial solid-phase double- antibody (Abbott Laboratories, Ill).

Specimens were cytopathologically analyzed for the presence of mucinous epithelium, extent of cytologic atypia and presence of malignant cells. A cellblock was prepared for further immunohistological staining, as described previously^27^.

The string-sign was determined at aspiration, by the endosonographer, as described previously^2^. In brief, a drop of fluid was placed between the thumb and index finger and the maximum length of stretch before disruption of the mucous string, was measured and recorded.

### Rheological measurements

PCF samples were stored at 4 °C, incubated at room temperature for one hour prior to measurements, and vortexed for 30 sec just before testing, to ensure homogenization.

Use of a rotational viscometer supports simulation of true rheological conditions (the stepping change of either the shear stress or the shear rate is programmed but the parameter remains constant during each step). The viscosity of the PCF samples was measured with a DHR-2 Rheometer (TA Instruments, USA) at 25 °C. The preferred geometry was cone-and-plate, with a cone diameter of 40 mm and a surface-plate angle of 1°. The rheometer was operated in shear rate control mode. Several time sweep tests at different constant shear rates (5–2000 1/s) were performed. The measured steady-state shear viscosity values (when the viscosity was constant in time) were used to construct flow curves of the fluids. Measurements were repeated 1–4 times for each sample, depending on the amount of fluid aspirated. The flow curves (viscosity versus shear stress or shear rate), characterizing the viscous response of the fluid under the shear field over a wide range of conditions, were then graphed.

### Data Analysis

Statistical analysis was performed using the nonparametric Mann-Whitney U-test, Pearson’s chi-squared test and receiver operating characteristic (ROC) curve. Cutoff points of age, CEA and viscosity were evaluated by ROC curve analysis and Youden’s statistics. Sensitivity, specificity and accuracy were calculated. A p value < 0.05 was considered significant. All analyses were performed using SPSS 21.0 (IBM Corp., Armonk, NY, USA).

## Additional Information

**How to cite this article**: Khamaysi, I. *et al*. Differentiation of Pancreatic Cyst Types by Analysis of Rheological Behavior of Pancreatic Cyst Fluid. *Sci. Rep.* **7**, 45589; doi: 10.1038/srep45589 (2017).

**Publisher's note:** Springer Nature remains neutral with regard to jurisdictional claims in published maps and institutional affiliations.

## Supplementary Material

Supplementary Information

## Figures and Tables

**Figure 1:**
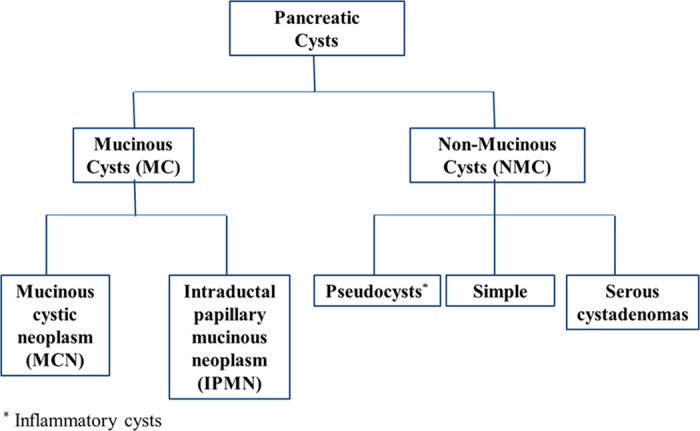
Classification of pancreatic cysts.

**Figure 2:**
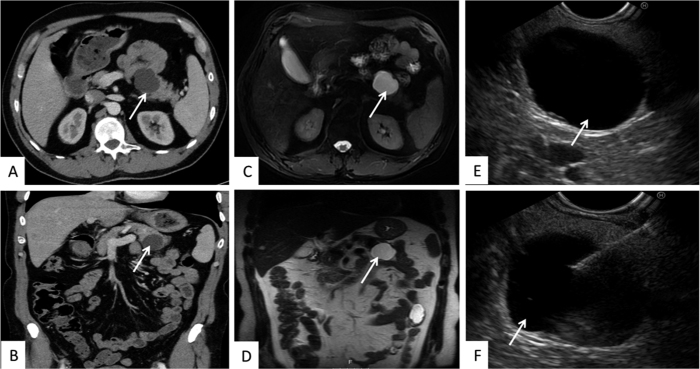
Pancreatic body cysts as detected by CT (**A,B**), MRI (**C,D**), EUS (**E**) and EUS-FNA (**F**). Arrows indicate the location of the pancreatic cyst.

**Table 1:** Patient demographics and cyst characteristics.

Sample no.	Sex	Age (y)	Cyst dia. (mm)	Location	CEA (ng/mL)	Amylase (U/L)	Cytology	String sign	Flow curve type	Final diagnosis	Cyst type
1	Female	46	28	Tail	531	22	Negative	0	I	SC	NMC
2	Male	47	22	Body	49.8	61	Negative	0	III	Simple	NMC
3	Female	75	25	Body	9776	29	Positive	1	II	IPMN	MC
4	Female	68	22	Body	286.4	2896	Negative	0	I	IPMN	MC
5	Female	53	70	Head	24808	67	Positive	1	III	Cancer	MC
6	Female	52	50	Head	0.1	67483	Negative	0	I	SC	NMC
7	Female	21	35	Body	0.1	64	Negative	1	II	SC	NMC
8	Male	21	30	Head	0.1	68	Negative	1	II	SC	NMC
9	Female	59	33	Body	0.1	111	Negative	1	II	SC	NMC
10	Male	77	35	Head	2324	7060	Negative	1	III	IPMN	MC
11	Female	39	35	Body	22.2	107580	Negative	0	II	PC	PC
12	Male	53	26	Body	5.1	282240	Negative	0	II	PC	PC
13	Male	62	20	Body	32.8	27018	Positive	1	II	IPMN	MC
14	Female	58	45	Body	29	50162	Negative	0	II	PC	PC
15	Male	77	30	Body	2.4	118000	Negative	0	III	IPMN	MC
16	Male	53	35	Body	12.4	465	Negative	0	II	SC	NMC
17	Female	70	70	Tail	1.2	60	Negative	0	III	Cancer	MC
18	Male	55	70	Tail	1282	3833	Negative	0	II	PC	PC
19	Male	58	60	Body	18	184000	Negative	1	II	PC	PC
20	Male	56	40	Body	3271	491	Negative	0	III	Cancer	MC
21	Female	78	80	Tail	103970	33	Positive	0	III	Cancer	MC
22	Male	78	30	Body	7.6	737000	Negative	1	III	IPMN	MC

SC - serous cystadenomas; MC - mucinous cyst; NMC - non-mucinous cyst; PC - pseudocyst; IPMN - intraductal papillary mucinous neoplasm. String sign = ‘1’, when there is a high likelihood of a mucinous cyst. Cytology was considered positive if the cytopathologist report included malignant (or suspicious) cells; otherwise, it was considered negative.

**Table 2:** Averaged values of the model parameters for the PCF samples.

Flow curve type #	*η*_*∞*_ (cP)	*K*	*n*
I	1.04 ± 0.10	<10^−5^	≈0
II	1.01 ± 0.13	0.025 ± 0.018	−0.79 ± 0.25
III	1.55 ± 0.26	0.026 ± 0.019	−0.62 ± 0.17

**Table 3:** Mean values of predictive factors in PCF sample subgroups.

	Flow curve	Number	Mean ± SD	Median (range)	P value
**Age (60 y)**		22 (total)	57.1 ± 16.2	57 (21–78)	
	*I*	3	55.3 ± 11.4	52 (46–68)	
	*II*	11	50.4 ± 16.8	55 (21–75)	
	*I* + *II*	14	51.4 ± 15.5	54 (21–75)	0.020
	*III*	8	67 ± 12.9	73.5 (47–78)	
**Amylase (U/L)**		22 (total)	72216 ± 165518	1694 (22–737000)	
	*I*	3	23467 ± 38146	2896 (22–67483)	
	*II*	11	59597 ± 94488	3833 (29–282240)	
	*I* + *II*	14	51855 ± 85605	3365 (22–282240)	0.297
	*III*	8	107847 ± 257487	279 (33–737000)	
**CEA (ng/mL)**		22 (total)	6656 ± 22429	26 (0–103970)	
	*I*	3	273 ± 266	286 (0–531)	
	*II*	11	1016 ± 2930	18 (0–9776)	
	*I* + *II*	14	857 ± 2591	20 (0–9776)	0.014
	*III*	8	16804 ± 36218	1187 (1–103970)	
**Cyst Diameter (mm)**		22 (total)	40.5 ± 18.1	35 (20–80)	
	*I*	3	33.33 ± 14.74	28 (22–50)	
	*II*	11	37.64 ± 15.19	35 (20–70)	
	*I* + *II*	14	36.71 ± 14.64	34 (20–70)	0.294
	*III*	8	47.13 ± 22.5	37.5 (22–80)	
**Viscosity,** *η*_*c*_ **(cP)**		22 (total)	1.32 ± 0.33	1.19 (0.97–2.08)	
	*I*	3	1.04 ± 0.1	0.99 (0.97–1.15)	
	*II*	11	1.13 ± 0.08	1.15 (1.01–1.24)	
	*I* + *II*	14	1.11 ± 0.09	1.15 (0.97–1.24)	<0.0001
	*III*	8	1.69 ± 0.26	1.64 (1.41–2.08)	

*η*_*c*_-Viscosity measured at strain rate 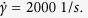

**Table 4:** Statistical parameters of various diagnostic approaches for differentiating between MC versus NMC lesions.

Method	Sensitivity	Specificity	PPV	NPV	Accuracy	P value
Age	0.800	1.00	1.00	0.857	0.909	0.000
String-Sign	0.500	0.667	0.556	0.615	0.591	0.666
CEA (192 ng/mL)	0.600	0.833	0.750	0.714	0.727	0.074
CEA (1000 ng/mL)	0.500	0.917	0.833	0.688	0.727	0.056
Amylase (1200 U/L)	0.500	0.500	0.455	0.545	0.500	1.000
PC/NMC- Amylase (1200 U/L)	1.00	0.857	1.00	0.857	0.917	0.015
Cytology	0.400	1.00	1.00	0.667	0.727	0.029
Viscosity, *η*_*c*_	0.700	0.917	0.875	0.786	0.818	0.006
**Combinations**						
CEA (192 ng/mL) + Amylase (1200 U/L)	0.200	0.917	0.667	0.579	0.591	0.571
CEA (192 ng/mL) + Amylase (1200 U/L) + string sign	0.600	0.833	0.750	0.714	0.727	0.074
string sign + CEA (192 ng/mL)	0.700	0.583	0.583	0.700	0.636	0.231
Viscosity, *η*_*c*_ + CEA (192 ng/mL)	0.900	0.750	0.900	0.900	0.818	0.004
Viscosity, *η*_*c*_ + Age (I + II & < 60)	1.00	0.917	1.00	1.00	0.955	0.000
Viscosity, *η*_*c*_ + CEA (1000 ng/mL)	0.800	0.833	0.800	0.833	0.818	0.008

PPV positive predictive value, NPV negative predictive value.

*η*_*c*_ - Viscosity measured at strain rate 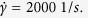
