# Supplementary material for: Differentiation of Pancreatic Cyst Types by Analysis of Rheological Behavior of Pancreatic Cyst Fluid
Source: Sci Rep. 2017 Mar 30;7:45589. doi: 10.1038/srep45589 (PMC5372360; doi:10.1038/srep45589)
Supplement: Supplementary Information [file srep45589-s1.doc]

Differentiation of Pancreatic Cyst Types by Analysis of Rheological Behavior of Pancreatic Cyst Fluid

*Iyad Khamaysi ^&^, Aiman Abu Ammar ^&^, Gleb Vasilyev, Arkadii Arinstein,* *Yehuda Chowers, Eyal Zussman*

**Supplementary Information**

**Table S1.** Patient age and various features of PCF samples

|  |  | **Number** | **Mean±SD** | **Median (range)** | **P value** |
| --- | --- | --- | --- | --- | --- |
| **Age** |  | 22 (total) | 57.1±16.2 | 57 (21-78) |  |
|  | *MC* | 10 | 69.4±9.4 | 72.5 (53-78) | 0.001 |
|  | *NMC* | 12 | 46.8±13.4 | 52.5 (21-59) |  |
|  | *NMC (except PC)* | 7 | 42.7±15.4 | 47 (21-59) | 0.192 |
|  | *PC* | 5 | 52.6±7.9 | 55 (39-58) |  |
| **Amylase (U/L)** |  | 22 (total) | 72216±165518 | 1694 (22-737000) |  |
|  | *MC* | 10 | 89265±230506 | 1694 (29-737000) | 0.692 |
|  | *NMC* | 12 | 58007±91351 | 2149 (22-282240) |  |
|  | *NMC (except PC)* | 7 | 9753±25457 | 68 (22-67483) | 0.012 |
|  | *PC* | 5 | 125563±110442 | 107580 (3833-282240) |  |
| **CEA (ng/mL)** |  | 22 (total) | 6656±22429 | 26 (0-103970) |  |
|  | *MC* | 10 | 14448±32403 | 1305 (1-103970) | 0.034 |
|  | *NMC* | 12 | 162±383 | 15 (0-1282) |  |
|  | *NMC (except PC)* | 7 | 85±198 | 0 (0-531) | 0.160 |
|  | *PC* | 5 | 271±565 | 22 (5-1282) |  |
| **Cyst Diameter (mm)** |  | 22 (total) | 41±18 | 35 (20-80) |  |
|  | *MC* | 10 | 42±22 | 33 (20-80) | 0.895 |
|  | *NMC* | 12 | 39±14 | 35 (22-70) |  |
|  | *NMC (except PC)* | 7 | 33.29±8.67 | 33 (22-50) | 0.164 |
|  | *PC* | 5 | 47.20±17.94 | 45 (26-70) |  |
| **Viscosity,** 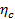**^a^** |  | 22 (total) | 1.32±0.33 | 1.19 (0.97-2.08) |  |
|  | *MC* | 10 | 1.54±0.37 | 1.53 (0.99-2.08) | 0.012 |
|  | *NMC* | 12 | 1.14±0.14 | 1.15 (0.97-1.47) |  |
|  | *NMC (except PC)* | 7 | 1.12±0.17 | 1.05 (0.97-1.47) | 0.088 |
|  | *PC* | 5 | 1.17±0.07 | 1.17 (1.06-1.24) |  |

^a^ Viscosity measured at strain rate
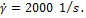


**Table S2.** Fitting parameters of the power law model for all tested PCFs

| **Flow curve type #** | **PCF sample #** | 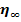**,** cP | ***K*** | ***n*** |
| --- | --- | --- | --- | --- |
| I | 1 | 1.16 | 1.52×10^-9^ | -0.002797 |
|  | 4 | 0.99 | 9.9×10^-6^ | -0.000409 |
|  | 6 | 0.97 | 9.9×10^-6^ | -0.000409 |
| II | 3 | 0.85 | 0.0019 | -0.177 |
|  | 7 | 0.91 | 0.0278 | -0.775 |
|  | 8 | 0.92 | 0.0143 | -0.785 |
|  | 9 | 0.99 | 0.0203 | -1.175 |
|  | 11 | 1.00 | 0.0394 | -0.712 |
|  | 12 | 0.80 | 0.0149 | -0.484 |
|  | 13 | 1.14 | 0.0127 | -1.028 |
|  | 14 | 0.98 | 0.0203 | -0.736 |
|  | 16 | 1.05 | 0.0288 | -0.781 |
|  | 18 | 1.19 | 0.0129 | -0.858 |
|  | 19 | 1.04 | 0.0716 | -0.863 |
|  | 22 | 1.22 | 0.0305 | -0.612 |
| III | 2 | 1.34 | 0.0033 | -0.412 |
|  | 5 | 1.41 | 0.0355 | -0.693 |
|  | 10 | 1.57 | 0.0105 | -0.435 |
|  | 15 | 1.33 | 0.0431 | -0.638 |
|  | 17 | 1.88 | 0.0423 | -0.896 |
|  | 20 | 1.36 | 0.0026 | -0.521 |
|  | 21 | 1.94 | 0.0456 | -0.727 |

**Table S3.** Patients (nos. 1,5,15, and 21) Cytological and Pathological findings

Case no. 1:

Imaging: Pancreatic tail cyst

EUS-FNA Cytology: Amorphous material, acellular, negative for malignancy

Cyst fluid analysis: High CEA, low amylase levels

Clinical Diagnosis: Mucinous cystadenoma (MC)

Surgical pathology: Serous cystadenoma (SC)

Case no. 5:

Imaging: Pancreatic head cyst

EUS-FNA Cytology: Moderately differentiated ductal adenocarcinoma

Cyst fluid analysis: High CEA, low amylase levels

Clinical Diagnosis: Pancreatic cystic adenocarcinoma

Surgical pathology: Adenocarcinoma (mucus producing), pancreatic head and liver metastasis

Case no. 15:

Imaging: Pancreatic cyst

EUS-FNA Cytology: Cyst wall with epithelial lining, macrophages and lymphocytes were observed.

Cyst fluid analysis: Low CEA, high amylase levels

Clinical Diagnosis: IPMN (MC)

Case no. 21:

Imaging: Complex pancreatic tail cyst

EUS-FNA Cytology: Necrotic material and well differentiated ductal adenocarcinoma malignant cells.

Cyst fluid analysis: High CEA, low amylase levels

Clinical Diagnosis: Pancreatic cystic adenocarcinoma

**Figure S1.** Flow curves of the PCFs (22 samples) at 25 °C.
